# Water Networks in Complexes between Proteins and FDA-Approved Drugs

**DOI:** 10.1021/acs.jcim.2c01225

**Published:** 2022-12-05

**Authors:** Marley L. Samways, Hannah E. Bruce Macdonald, Richard D. Taylor, Jonathan W. Essex

**Affiliations:** † School of Chemistry, University of Southampton, Southampton SO17 1BJ, U.K.; ‡ Computational and Systems Biology Program, Memorial Sloan Kettering Cancer Center, New York, New York 10065, United States; § UCB, 216 Bath Road, Slough SL1 3WE, U.K.

## Abstract

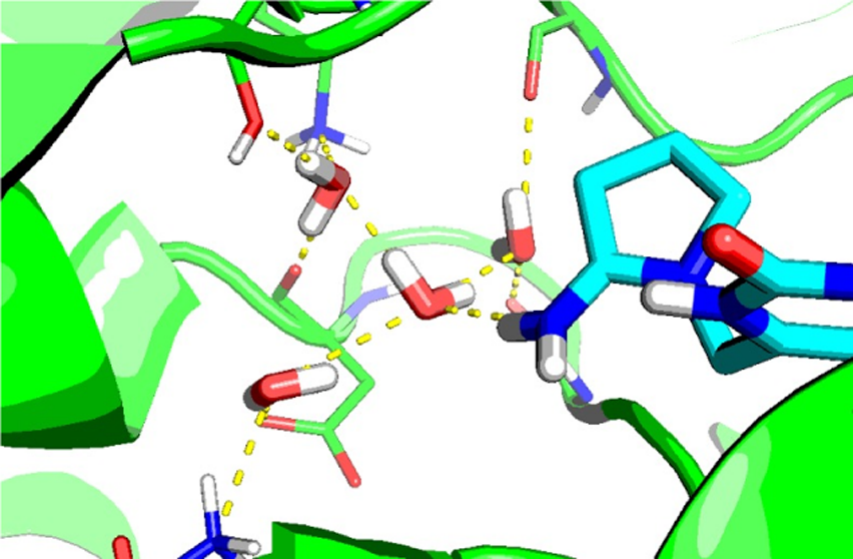

Water molecules at protein–ligand interfaces are often of significant pharmaceutical interest, owing in part to the entropy which can be released upon the displacement of an ordered water by a therapeutic compound. Protein structures may not, however, completely resolve all critical bound water molecules, or there may be no experimental data available. As such, predicting the location of water molecules in the absence of a crystal structure is important in the context of rational drug design. Grand canonical Monte Carlo (GCMC) is a computational technique that is gaining popularity for the simulation of buried water sites. In this work, we assess the ability of GCMC to accurately predict water binding locations, using a dataset that we have curated, containing 108 unique structures of complexes between proteins and Food and Drug Administration (FDA)-approved small-molecule drugs. We show that GCMC correctly predicts 81.4% of nonbulk crystallographic water sites to within 1.4 Å. However, our analysis demonstrates that the reported performance of water prediction methods is highly sensitive to the way in which the performance is measured. We also find that crystallographic water sites with more protein/ligand hydrogen bonds and stronger electron density are more reliably predicted by GCMC. An analysis of water networks revealed that more than half of the structures contain at least one ligand-contacting water network. In these cases, displacement of a water site by a ligand modification might yield unexpected results if the larger network is destabilized. Cooperative effects between waters should therefore be explicitly considered in structure-based drug design.

## Introduction

Water molecules that are in contact with protein are typically much more restricted in terms of their translational and rotational motions than bulk water. Accordingly, when a water is released from a constrained environment into bulk solution, there is an increase in entropy^1^—this can contribute favorably to the binding affinity of a ligand.^2−6^ As such, protein-bound waters are now a widely recognized feature of structure-based drug design.^7−9^ Targets where significant boosts in affinity have been associated with water displacement include HIV protease,^10^ neuraminidase,^11^ and BACE-1,^12^ among many others. It is important to note that the entropic benefit of water displacement may not be realized if the ligand fails to adequately recover the enthalpic interactions made by the water with the protein^13^—in such cases, water displacement can negatively impact the binding affinity of the ligand.^14,15^ It is rarely clear a priori whether the affinity of a particular compound would be best improved by the displacement or stabilization of a given water site. However, prior to making this decision, it is first important for the researcher to identify where water molecules are likely to bind at the protein–ligand interface so that they can be factored into structure–activity relationship (SAR) analyses.

X-ray crystallography is by far the most widely used experimental method for the structural analysis of protein-bound water molecules.^16^ However, this method carries some limitations regarding the identification of water molecules—notably, the electron density can be poorly resolved if the site is disordered, and the electron density can be confused with that of isoelectronic ions.^16−18^ In addition, hydrogen atoms are not resolved, which can cause ambiguity of the donor/acceptor partners in hydrogen-bonding interactions. For these reasons, a number of computational methods have been developed to identify water-binding sites.^19^ Broadly, these methods include knowledge-based methods, which extract hydration patterns from crystal structure data, and extrapolate these patterns to new structures;^20−28^ interaction-based prediction methods, which attempt to identify stable water binding sites, based on a search of possible water binding sites, coupled with a model of the intermolecular interactions;^29−35^ and more expensive simulations can be performed, typically using more complex energy models (known as force fields), from which the locations sampled by waters can be extracted.^36−43^ These computational methods have several benefits over X-ray crystallography: they can typically provide water predictions much more rapidly, and less resource-intensively than the solution of a crystal structure; they can often identify more disordered water sites, which would be poorly resolved experimentally; and they can also be applied easily to proteins which are not easily crystallized (such as membrane proteins, for example). However, it should be noted that these methods require a structure of the complex—in a prospective study, this might be obtained from crystal structures of similar complexes or by homology modeling. These methods are often assessed by their ability to reproduce crystallographic water sites. It should also be noted that many of these methods predict water sites independently of one another—this could be problematic in the event that cooperative effects between waters play a significant role. These methods are discussed in depth in our recently published review.^19^

The choice of protein–ligand crystal structures used to parameterize and assess the quality of these methods is critical. A recent study has demonstrated that the datasets used are often small, contain older structures, are strongly biased to particular proteins, and are not pharmaceutically relevant.^19^ Here, we seek to address this issue by creating a rigorously curated dataset of crystal structures containing Food and Drug Administration (FDA)-approved drugs.

Grand canonical Monte Carlo (GCMC) is a rigorous simulation technique that can be used for the enhanced sampling of buried water sites.^44−51^ The molecular simulation is performed under conditions of constant chemical potential (μ), volume, and temperature. Monte Carlo moves are carried out in which the insertion and deletion of waters to/from a defined region of interest^46,47^ (GCMC box) are attempted, allowing water molecules to rapidly bind and unbind to/from the protein binding site. The balance between the probabilities of binding and unbinding is determined by the Adams parameter^52,53^ (*B*), which is directly related to the chemical potential of the system. The *B*-value which gives an equilibrium between the GCMC box and bulk water (*B*_equil_) can be trivially computed^47^
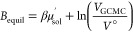
1where *V*_GCMC_ is the volume of the GCMC region, μ′_sol_ is the excess chemical potential of bulk water, and *V*° is the standard state volume of bulk water—these latter two parameters are taken as −6.2 kcal mol^–1^ and 30 Å^3^, respectively, as determined in previous work.^46^ It should also be noted that cooperative effects between water molecules are captured implicitly by GCMC.^46,47^

Past work has shown that GCMC simulations perform very well in terms of the prediction of water-binding sites, but these tests have been limited to a very small number of systems.^46,54,55^ In this work, we present a new dataset for testing water predictions, consisting of 108 complexes between proteins and small-molecule drugs. We carry out a much more extensive test of the ability of GCMC to predict crystallographic water binding locations, and also investigate the factors that impact the accuracy of the predictions. Given the ability of GCMC to capture cooperative effects between water molecules, we also provide an analysis of the water networks found within protein–ligand binding sites and discuss their implications for drug design.

## Methods

### Dataset Curation

A list of all drugs in the FDA Orange Book (as of July 2017) was assembled. Several conditions were imposed to triage the list of drugs: no fewer than five carbon atoms; no phosphorous atoms; molecular weight between 100 and 750 Da; fewer than 10 rotatable bonds; fewer than 10 atoms in a single ring. This was intended to restrict the drugs to small molecules which are not overly problematic for simulation. From this filter, 279 compounds were left, corresponding to 1554 structures in the Protein Data Bank (PDB).^56,57^

A second round of filtering was then applied to the PDB entries to eliminate low-resolution crystal structures and those containing features that are not easily treated by classical simulation methods. All structures released prior to 2000 or with a resolution worse than 2.5 Å were discarded, with the additional restriction that all structures must be of human, bacterial or viral origin. Structures containing no water molecules were excluded, along with those with any missing residues in close proximity to the ligand, and structures with close contacts for the asymmetric unit. If the protein–ligand interface showed covalent binding, co-binding molecules, or metal ions, the structure was also discarded. The remaining structures were further triaged such that no drug or protein was repeated more than five times, and no drug–protein pair was repeated. Two structures were then removed, as their PDB entries did not include electron density maps. leaving 105 structures.

**Figure 1:**
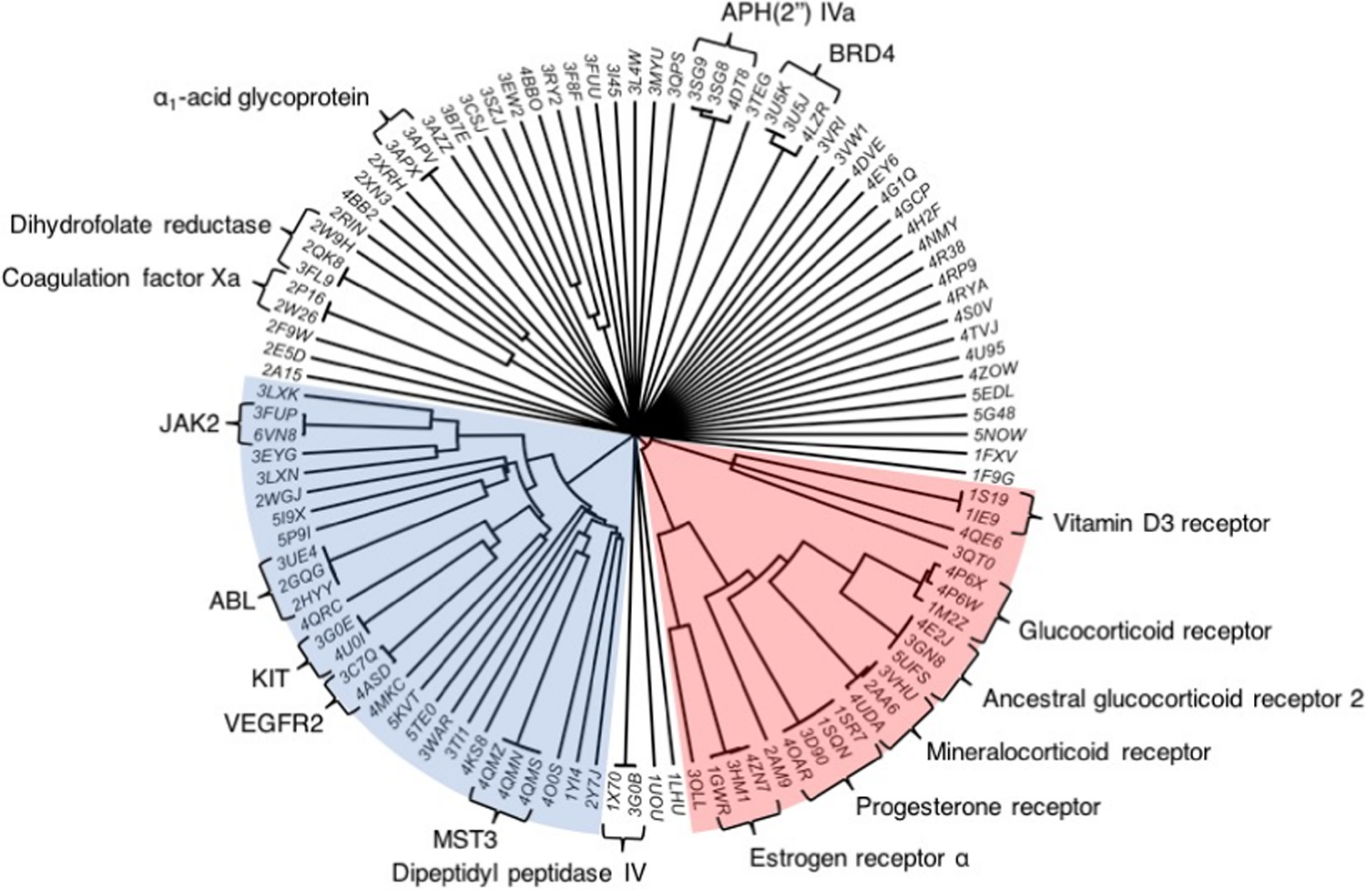
Phylogenetic tree of all proteins from the dataset curated in this work. The structures highlighted in blue and red correspond to kinases and nuclear receptors. BRD4: bromodomain-containing protein 4. APH(2″) IVa: aminoglycoside-2′′-phosphotransferase type IVa. VEGFR2: vascular endothelial growth factor receptor 2. MST3: mammalian sterile 20-like kinase 3. JAK2: Janus kinase 2.

In July 2022, the above was repeated for drugs released in or after 2017, leading to an additional three structures, resulting in a final dataset of 108 unique drug–protein crystal structures. A phylogenetic tree of this dataset is shown in Figure 1, and the PDB codes are listed in full in Table S1.

### Simulation Details

All protein–drug complexes were prepared using Maestro,^58^ followed by visual inspection of the protonation states and tautomers assigned. Protein scoops were created using a 30 Å distance threshold from the ligand atoms: residues within 15 Å of the ligand were sampled, those between 15 and 30 Å were constrained, and those beyond 30 Å were removed altogether—if a single atom from a residue is within a cutoff distance, the entire residue is considered to be within the cutoff. The scooped protein was then solvated in a spherical water droplet with a radius of 30 Å (waters were held within the droplet, using a half-harmonic restraint with a force constant of 1.5 kcal mol^–1^ Å^–2^). The proteins, ligands, and water were modeled using the AMBER ff14SB,^59^ GAFF14^60^ (with AM1-BCC charges^61,62^), and TIP4P^63^ force fields, respectively. All simulations were run at 298 K, using an interaction cutoff of 15 Å, with a switching function applied to the last 0.5 Å. The GCMC box was defined as a cuboidal region, extending at least 4 Å from all ligand heavy atoms—the coordinate frame of the system was rotated to minimize the volume of the GCMC box. All crystallographic water sites were removed prior to simulation, along with any water molecules located within the GCMC box.

Each system was then subjected to GCMC simulation in ProtoMS 3.4,^64^ at Adams values of *B*_equil_ – 0.5, *B*_equil_, and *B*_equil_ + 0.5. It should be noted that water sampling could be performed using just one simulation at *B*_equil_, but the additional simulations were used here to improve the sampling via replica exchange between adjacent *B*-values.^47^ Each simulation was first equilibrated for 10 million (10M) moves where only waters in the GCMC box were sampled, with moves split equally between insertions, deletions, and configurational sampling. A second equilibration stage of 10M moves allowed configurational sampling of the protein, ligand, and bulk solvent, with this sampling and that of the GCMC waters shared equally. This was continued for 40M moves of production, with coordinates saved and replica exchange moves attempted every 100k moves.

During the configurational sampling of the protein and ligand, all protein and ligand heavy atoms were constrained to their initial positions, to maximize the overlap between the simulated and crystallographic structures. Increased configurational sampling has been observed to have a negative impact on the comparison between simulated and crystallographic water sites.^65^

### Clustering Analysis

The water sites observed in each GCMC simulation were clustered, based on the locations of the oxygen atoms, using average-linkage hierarchical clustering (as implemented in SciPy^66^), with a distance cutoff of 2.4 Å. Waters present in the same simulation frame were assigned an arbitrarily high distance, to prevent them from being clustered together. The position of each cluster was taken as the closest constituent oxygen position to the cluster centroid observed. Each cluster obtained therefore has an associated position and occupancy (based on the number of waters in this cluster, relative to the number of simulation frames). The occupancy of a GCMC cluster is related to the stability of a water site in that location—a water site with a standard binding free energy of zero would be expected to be present for 50% of the simulation, as it would be equally stable in the binding site and bulk water. It should be noted that this clustering algorithm works best when there are well-defined peaks in the water density, and produces a large number of clusters in regions where the density is very diffuse (such as regions that are highly solvent-exposed).

### Water Network Analysis

We carried out the following analysis to extract water networks from the sets of water clusters for each system. Starting from a given water cluster, waters are iteratively added to the network if within hydrogen-bonding distance of any water already in the network—a hydrogen bond is counted as a distance of less than 3.2 Å between cluster centers. However, there are two conditions that will reject the addition of a water to the network. First, we impose that the occupancy of the network must be at least 50% (such that it is present more than it is absent), so if the addition of a water would reduce the occupancy below this threshold, then the water is not added. Second, the addition is rejected if the additional water is anticorrelated with any water already in the network—a pair of waters are considered anticorrelated if the percentage of frames in which they are found together is more than 10% less than the product of their occupancies. For example, two waters with occupancies of 50% would be expected to be found together in 25% of simulation frames if they were independent, therefore, if they are found together in fewer than 15% of simulation frames, their binding is considered to be anticooperative. Having built a set of networks, they are then filtered. First, we impose that all networks must contact the ligand—where at least one water in the network is within 3.4 Å of a ligand heavy atom—and any networks which do not satisfy this criterion are discarded. Finally, where any pair of networks contain a subset of the same waters, we discard the less-occupied network. A representative frame was then written out for each network.

## Results and Discussion

### Accuracy of the GCMC Water Predictions

As previously mentioned, the assessment of water predictions is typically carried out by a comparison of the predicted locations to crystallographic water sites. This assessment can be treated as a binary classification problem, where we employ the following definitions. A true positive (TP) indicates a predicted water site that matches an experimental site, a false positive (FP) is a predicted site that does not match an experimental site, and a false negative (FN) is a crystallographic site for which there is no predicted site. For this problem, the number of true negatives (TN)—where there is no experimental or predicted site—cannot be counted. In this work, we make use of two metrics for this analysis, the first of which is the true positive rate (TPR, or sensitivity)
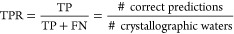
2which indicates the fraction of experimental sites which are correctly identified, and the second is the positive predictive value (PPV, or precision),
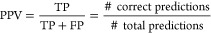
3which indicates the fraction of predicted sites that correspond to an experimental site.

To assess the quality of the predictions made for the clustered water sites extracted from GCMC simulations, we determine a predicted site to match an experimental site if their positions are within 1.4 Å of each other—the effect of this decision is discussed further below. Note that if the occupancy of an experimental water site is split over two positions, then only one of these will be used (that which more closely matches a predicted site). To exclude bulk water sites (which are of little interest) from this analysis, we restrict the classification to only those waters which have at least one nonwater hydrogen bond (throughout this work, a hydrogen bond is defined as a distance of 3.2 Å or less between polar heavy atoms). Additionally, as GCMC clusters with occupancies less than 50% are expected to bind unfavorably, these are also excluded. From this, we obtain a TPR of 0.814 and a PPV of 0.263. However, some of the predicted sites lie at the edges of the GCMC box and match crystallographic sites outside the box that were not considered—if these predicted sites are not counted as FPs, the PPV improves slightly to 0.283. The TPR appears rather good and is compared to those reported for other methods in the following section. It should be noted that while the PPV appears to indicate that the predictions are rather imprecise, this is common for simulation methods,^67^ as many of the disordered and solvent-exposed sites will be poorly resolved by X-ray crystallography.

### Factors Affecting TPR

**Figure 2:**
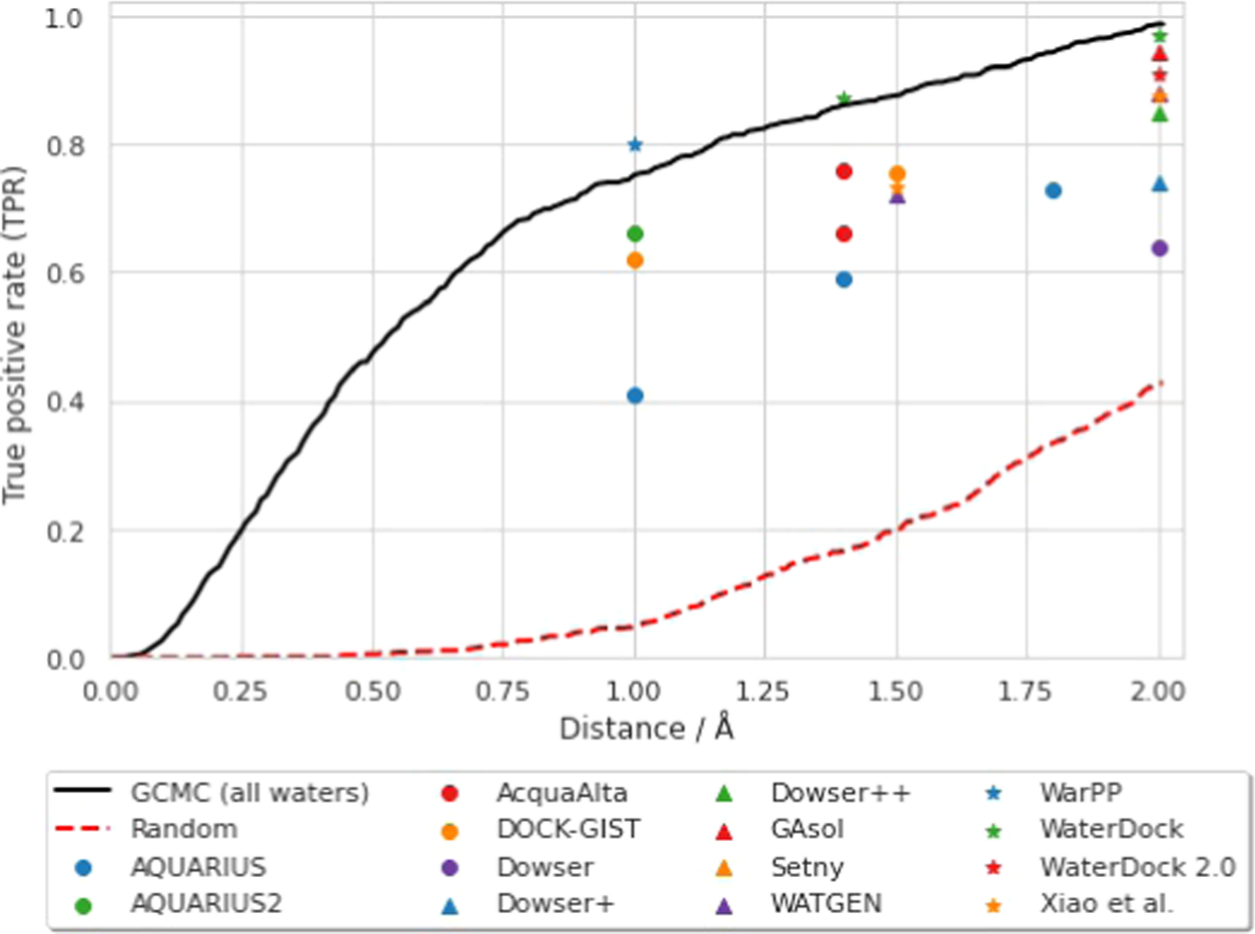
Graph showing the effect of the distance threshold on the TPR observed. The black line shows the results obtained in this work, when considering all crystallographic water sites (within the GCMC region for each structure) and all GCMC sites. The dashed red line shows the results from random placement of water molecules—for each structure an equivalent number of waters to the number of GCMC clusters were randomly placed within the GCMC volume. For comparison, we include TPR values reported at various distance thresholds by other methods (note that the datasets and selection criteria vary^19^): AQUARIUS,^20^ AQUARIUS2,^21^ AcquaAlta,^25^ DOCK-GIST,^68^ Dowser,^69^ Dowser+,^70^ Dowser++,^71^ GAsol,^35^ Setny,^72^ WATGEN,^67^ WarPP,^28^ WaterDock,^32^ WaterDock 2.0,^34^ and Xiao et al.^27^

In the above, several choices were made which could impact the assessment of the predictions, regarding the exclusion of certain water sites and the distance threshold at which predicted sites are considered to match crystallographic sites. First, we investigate the impact of the distance threshold, which is chosen somewhat arbitrarily. The dependence of the TPR on this parameter is plotted in Figure 2 (note that all water molecules are considered here), and published TPR values from other methods included for reference. The values of the TPR at different distances are given in Table S2. As might be expected, the TPR increases monotonically with the distance threshold, reaching a value of 0.986 at 2.0 Å. Comparison with the other reported methods indicates that the performance of GCMC is very competitive. However, it should be noted that this is not a like-for-like comparison, as the different values were reported on not only different datasets, but also different subsets of the water molecules within those datasets—for example, some methods consider only binding site waters, or may filter crystallographic waters by the number of hydrogen bonds^19^ (as described previously in this work).

**Figure 3:**
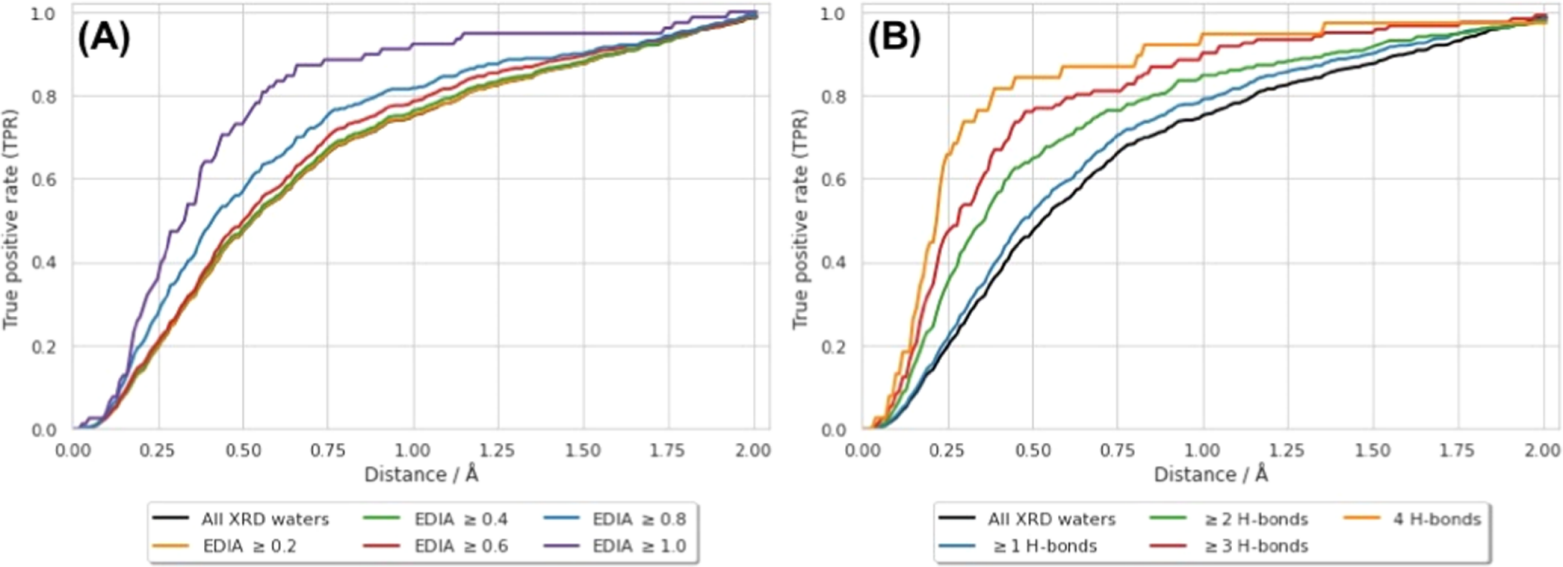
Graphs showing how the TPR is affected by the exclusion of crystallographic sites, which do not meet certain criteria. In each case, the black line is identical to that in Figure 2, for reference. (A) TPR vs distance curves for different EDIA thresholds, where only crystallographic waters with a score greater than, or equal to, the specified threshold are considered. (B) Similarly, curves are plotted for different thresholds of the number of nonwater hydrogen bonds made by the water sites.

Here, we investigate the other factors affecting whether crystallographic water sites are correctly identified by GCMC. First, we consider the electron density support for the crystallographic waters, quantified via the electron density for individual atoms (EDIA) score^73,74^ (calculated using the ProteinsPlus server^75,76^), which ranges from 0 to 1.2, with higher scores indicating a greater degree of electron density at the water location. Second, we consider the number of hydrogen bonds made by the water to nonwater atoms (note that this value is capped at 4, if the water oxygen is close to a large number of atoms). While it might be expected that these two variables are coupled, it transpires that they are poorly correlated (*R*^2^ = 0.05, Figure S1). These data are plotted in Figure 3, with the data also given in Tables S3 and S4.

Figure 3a shows that, for waters with higher EDIA scores, the TPR is increased at almost all distance thresholds. This indicates that crystallographic water sites which are better represented by the raw electron density are more reliably predicted by GCMC, which is reassuring. Waters with EDIA scores of 0.6 and below, show very similar TPR values. The improvement in TPR performance becomes notable for those waters with scores of 0.8 or better. Interestingly, a value of 0.8 or higher was suggested by Meyder et al. as strong evidence for the presence of a water site—those with scores between 0.4 and 0.8 are suggested to show minor inconsistencies with the electron density, and a score below 0.4 indicates major inconsistencies.^74^Figure 3b shows that the number of hydrogen bonds of a crystallographic water site also has a significant impact on how well the site is predicted. Notably, a significant improvement in the TPR curve is seen when the minimum number of hydrogen bonds is increased to 2. The increase in TPR with the number of hydrogen bonds likely reflects the fact that, for a larger number of hydrogen bonds with the protein/ligand, the water binding free energy is more negative and the water site more clearly defined. Conversely, if a water site has only one protein/ligand hydrogen bond, there are a larger number of similarly stable positions that the water might adopt, making it more difficult to yield the experimentally observed position—this increase in the positional disorder also makes it less likely that the site will be experimentally resolved.

These trends indicate that crystallographic water sites which have better electron density evidence, and show more hydrogen bonds with the protein–ligand complex, are more likely to be successfully reproduced by a GCMC simulation. However, these data also show that for the same set of structures, significantly different performances can be obtained from the same data by different filtering of the crystallographic water sites which are considered for prediction. For this reason, we have included a XLSX file in the Supporting Information, containing a list of the 723 experimental water sites considered in this work as a community test set.

### Factors Affecting PPV

**Figure 4:**
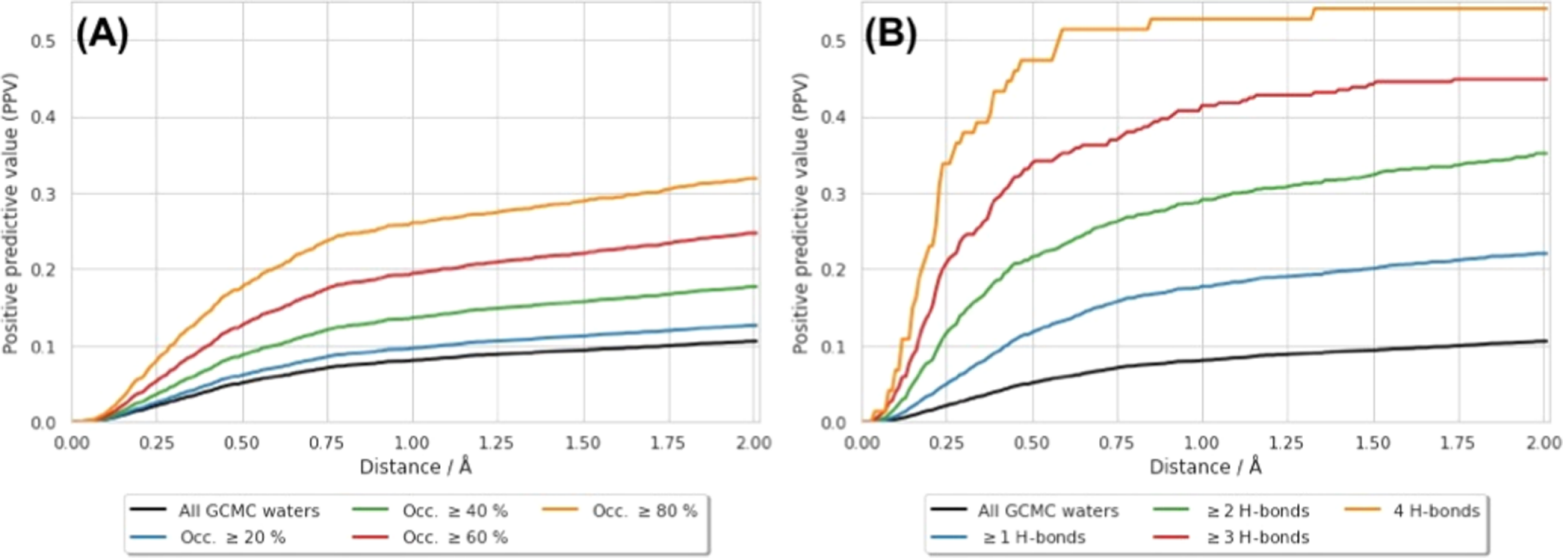
Graphs showing how the PPV is affected by different categories of GCMC sites. (A) PPV plotted against distance for different minimum thresholds of cluster occupancy. (B) Similar plot, where the GCMC sites are filtered based on the number of hydrogen bonds formed with protein/ligand atoms.

It is also of interest to carry out a similar analysis, to identify the factors which make a GCMC prediction more likely to correspond to a crystallographic site. This is especially important for prospective applications of water prediction methods, where one may not have access to the crystal structure for the specific protein–ligand complex of interest (when working from a homology or docking model, for example), and therefore needs to interpret which predicted water sites are more reliable. The two factors we consider for the GCMC sites are the occupancy of the cluster, and the number of protein/ligand hydrogen bonds—these descriptors are not orthogonal, but the correlation between them is weak (*R*^2^ = 0.21, Figure S2). Figure 4a shows a plot of the PPV against the distance threshold for different levels of cluster occupancy. This plot shows that restricting the analysis to higher occupancy waters improves the precision of the predictions. As previously mentioned, the occupancy of a site in a GCMC simulation is related to the stability of the site, and it therefore follows that more stable sites are more likely to be well resolved in a crystal structure. Figure 4b shows an analogous plot for different numbers of protein or ligand hydrogen bonds, where again this appears to be a significant factor. GCMC sites with more hydrogen bonds are therefore more likely to correspond to crystallographically identified sites. Conversely, sites with low occupancies and few hydrogen bonds to nonwater molecules are, understandably, less likely to be observed experimentally.

These observations are highly relevant, as in prospective applications, where water site predictions are necessary, there may not be any crystallographic data against which to assess the predicted water sites. It is therefore of use to a researcher employing GCMC to be able to distinguish the sites which are of greater significance from those which can be safely ignored. It should also be noted that the values of the PPV plotted in Figure 4 (and given in Tables S5 and S6) are inherently underestimated, as the crystal structure does not contain all water molecules which are truly present at the protein–ligand interface, owing to the previously described issues.

### Network Analysis

**Figure 5:**
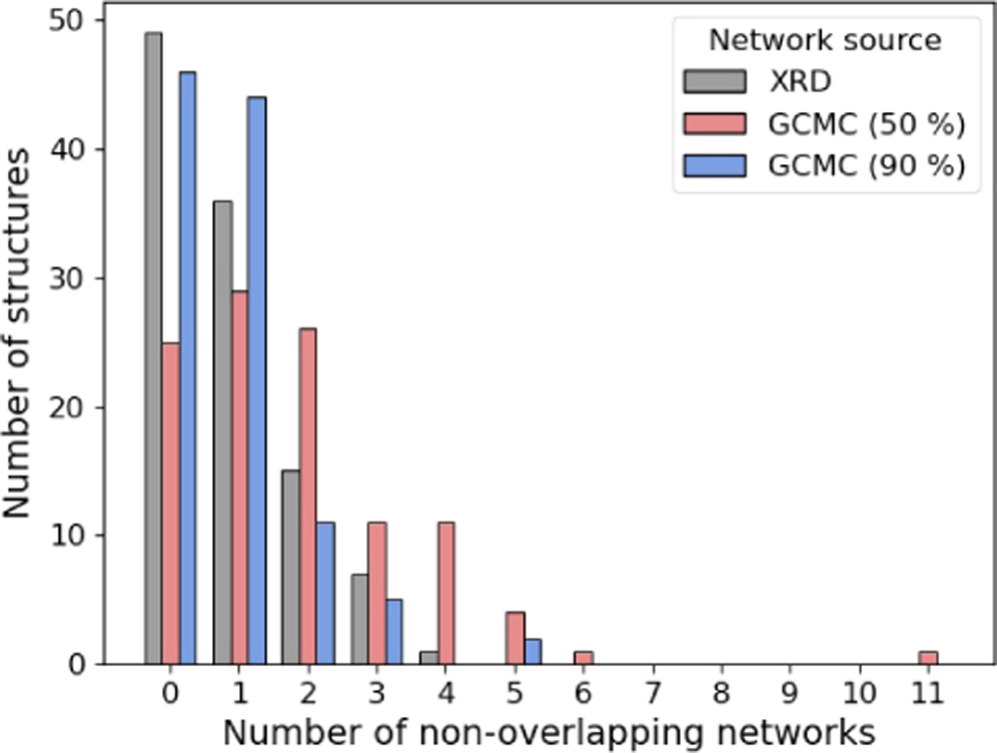
Bar chart showing the number of structures containing different numbers of water networks, as determined in the main text. Note that we only consider nonoverlapping water networks (i.e., that share no waters in common), where at least one water site is in contact with the ligand.

Having carried out the aforementioned network analysis, we find that 83 of 108 structures have at least one ligand-contacting water network which is present for at least 50% of the simulation—note that ligand-contacting networks are of particular interest, as they might be disturbed by ligand modifications. However, it should be noted that this figure is dependent on the chosen occupancy level of 50% (which was chosen somewhat arbitrarily), so the analysis was repeated with a much stricter occupancy criterion of 90%, where 62 of 108 structures still show at least one network. For reference, inspection of the crystallographic structures reveals that 59 of the structures exhibit at least one such water network. These results are shown in Figure 5, where it is clear that both crystallography and GCMC are in agreement that a large number of the complexes include at least one (and in many cases, more) water network in contact with the ligand—though there is some disagreement as to exactly how prevalent the networks are. In any case, the fact that more than half of these protein–drug complexes exhibit water networks in contact with the ligand highlights that water molecules should not be treated as independent entities in structure-based drug design. When considering ligand modifications that would displace a water molecule, researchers would be well advised to thoroughly consider the effects on any secondary water sites, which may be destabilized. This effect has been previously reported in free energy calculations of water networks.^47^ Importantly, the GCMC data appear to indicate that these effects are more common than would be inferred from the crystallographic data as the sites of noncontact waters are less likely to be resolved in a crystal structure.

**Figure 6:**
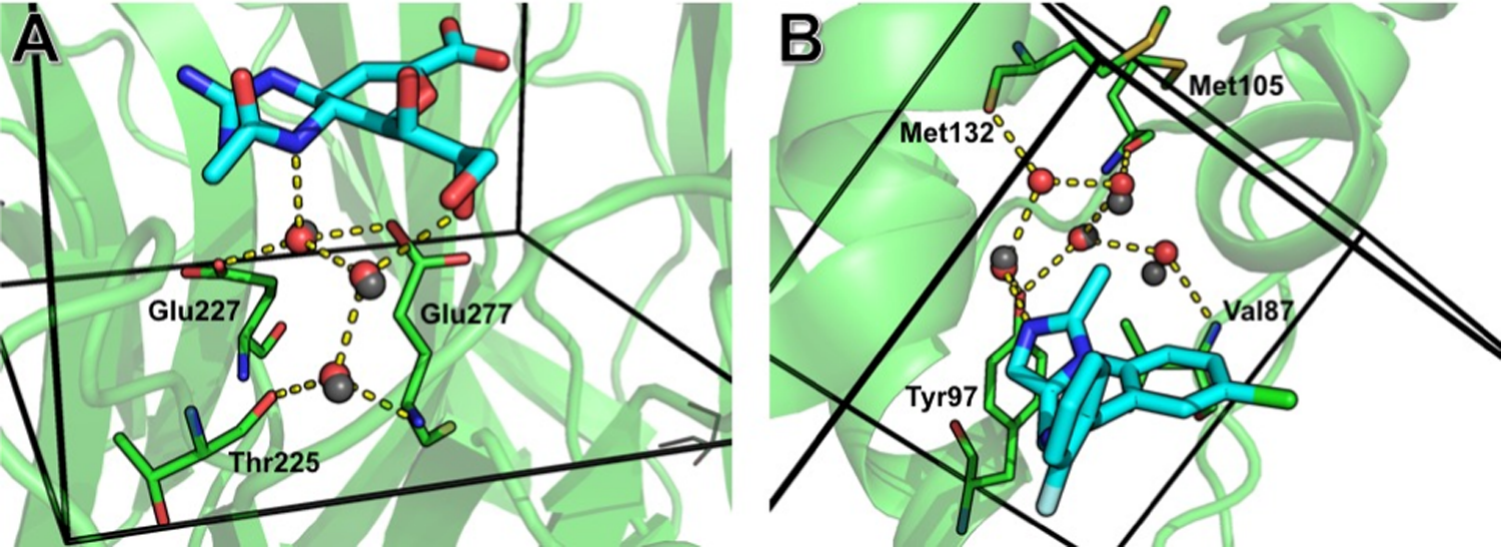
Two examples of water networks identified in this work. The waters shown as red spheres were identified from the GCMC network analysis described in the main text, using a minimum network occupancy of 90%—hydrogen bonds involving these waters are indicated with dashed yellow lines. Solid black lines indicate the limits of the GCMC box, and relevant crystallographic water sites are shown as gray spheres. Protein residues making hydrogen bonds with the water network are shown as green sticks and labeled. (A) Zanamivir bound to neuraminidase (PDB ID: 3B7E), showing a three-water network with an occupancy of 100%. (B) Midazolam in complex with BRD4 (PDB ID: 3U5K), showing a five-water network with an occupancy of 99%.

Figure 6 shows two examples of the networks identified using this approach, where cooperative effects between waters might be expected to complicate water displacement. Figure 6A shows zanamivir in complex with neuraminidase, where we identified a three-water network in 100% of simulation frames, indicating that the network is very stable. Figure 6B shows midazolam in complex with BRD4 (note that water networks have been well studied for bromodomains^77^), where a network of five water molecules was identified in 99% of simulation frames. Four out of five of these waters are observed crystallographically, although it is interesting to note that the fifth water is also observed in other binding sites within the same asymmetric unit of this PDB entry. Nonetheless, this example demonstrates how simulations can complement experimental data.

## Conclusions

We have presented a large-scale analysis of the performance of grand canonical Monte Carlo (GCMC) simulations for the prediction of water molecules in protein–ligand binding sites. For this work, we curated a novel dataset of 108 protein–ligand crystal structures (Table S1), where all ligands are FDA-approved drugs. All structures in this dataset were released in or after the year 2000 and have a resolution better than, or equal to, 2.5 Å. Water locations in these binding sites were predicted by clustering the positions observed in GCMC simulations. We find that 81.4% of nonbulk crystallographic water sites (those with at least one protein/ligand hydrogen bond) are correctly identified by GCMC to within 1.4 Å. However, we find that this performance is very sensitive to the specific success criteria.

As might be expected, the number of crystallographic waters reproduced is very dependent on the distance threshold used to define a successful prediction (Figure 2). While investigating the factors which separate the crystallographic waters which are well predicted from those which are not, we identified two trends: That crystallographic waters are more likely to be predicted when they are better supported by the underlying electron density (measured via the EDIA score^73,74^), and also when they exhibit more hydrogen bonds with the protein and/or ligand. However, it is not uncommon for researchers to exclude crystallographic sites which do not surpass some thresholds of EDIA score and/or number of hydrogen bonds when assessing the accuracy of a prediction method.^27,28^ Our analysis suggests that these decisions could unintentionally have a significant impact on the reported performance of a prediction method, making it very difficult to fairly compare reported results from different methods. Differences in benchmark systems used and in the assessment criteria could therefore both obscure the true performance of these methods. Thus, to facilitate comparison with our results by other groups, we include in the Supporting Information a XLSX file containing the details of the crystallographic waters considered in this work, a ZIP file containing the prepared protein structures used (such that interested readers can verify the protonation states simulated in this work, and the residues included in the protein scoop), and also the numerical values of the performance measured under the different criteria discussed (Tables S2–S6). We hope that the dataset curated in this work proves useful in this regard, but the field would also benefit from a blind challenge, where water predictions for a series of structures might be submitted to an independent party for analysis.

It should also be noted that there are two other factors that can affect the comparison between simulated and experimental results which have not been discussed here. First, most of the crystal structures considered were collected at cryogenic temperatures, whereas the simulations were performed at room temperature. The significant difference in temperature may have an impact on the stabilities of some of the water sites, and could potentially be the cause of some of the discrepancies observed. Second, it is not clear to what extent the interaction model (force field) causes disagreement between simulation and experiment—one can imagine situations where inaccuracies in the energy calculations might cause particular water sites to be over- or understabilized in the simulation. Notably, force fields for water are typically parametrized to model bulk water—these parameters may be inappropriate for protein binding sites, where the environment is likely to polarize waters differently to bulk water. Future work which accounts for polarization in the prediction of water-binding sites would be very interesting.

In addition, we have presented an analysis of the water networks found in this dataset. Both crystallographic and simulation data indicate that ligand-contacting water networks are very common. The simulation data tend to indicate that these are more common than would be inferred from the crystallographic data alone—likely because waters which do not directly contact the protein/ligand are less likely to be crystallographically resolved. In any case, this observation has important implications for drug design. Often, analyses of interfacial waters consider them in isolation of other water sites—our results suggest that this is often inappropriate, and that cooperative effects between water molecules should not be neglected when targeting them for displacement.

## Supporting Information Available

Dataset curated for analysis in this work (Table S1); numerical data showing the TPR values obtained at different distance thresholds (Tables S2–S5); numerical data showing the PPV values obtained at different distance thresholds (Table S6); and plot of the number of protein/ligand hydrogen bonds made (Figures S1 and S2) (PDF)
List of crystallographic waters considered (XLSX)

## Notes

The initial structures used to prepare each simulation were obtained from the Protein Data Bank, where the identification codes are given in the Supporting Information. The prepared protein–ligand structures used for simulation are made available in the Supporting Information. The workflows followed are described in the Methods section. The EDIA scores for the water molecules were obtained from the ProteinsPlus server (www.proteins.plus). The ProtoMS software can be downloaded free of charge from www.protoms.org. Prepared system files, as simulated in this work. This material is available free of charge via the internet at https://doi.org/10.5281/zenodo.7124390.

## Abbreviations

PDB: Protein Data Bank
GCMC: grand canonical Monte Carlo
TP: true positive
FP: false positive
FN: false negative
TPR: true positive rate
PPV: positive predictive value
