# Supplementary material for: Water Networks in Complexes between Proteins and FDA-Approved Drugs
Source: J Chem Inf Model. 2022 Dec 5;63(1):387–96. doi: 10.1021/acs.jcim.2c01225 (PMC9832485; doi:10.1021/acs.jcim.2c01225)
Supplement: Supplementary file 1 — ci2c01225_si_001.pdf [file ci2c01225_si_001.pdf]

Supporting Information for “Water networks in complexes between proteins and FDA-approved drugs”

Marley L. Samways[a], Hannah E. Bruce Macdonald[b], Richard D. Taylor*[c], Jonathan W. Essex*[a]

a) *School of Chemistry, University of Southampton, SO17 1BJ, UK*
b) *Computational and Systems Biology Program, Memorial Sloan Kettering Cancer Center, New York, New York, 10065, USA*
c) *UCB, 216 Bath Road, Slough, SL1 3WE, UK*

Email: rich.taylor@ucb.com, j.w.essex@soton.ac.uk

Supplementary Tables

Table S1. Dataset curated for analysis in this work. For each structure, the following information is given: Protein Data Bank (PDB) ID, source organism, protein name, drug name, structure resolution, and release year.

PDB ID	Organism	Protein	Drug	Res. / Å	Year
1F9G	*S. pneumoniae*	Hyaluronate lyase	Ascorbic acid	2.00	2001
1FXV	*E. coli*	Penicillin acylase	Penicillin G	2.25	2001
1GWR	*H. sapiens*	Estrogen receptor alpha	Estradiol	2.40	2002
1IE9	*H. sapiens*	Vitamin D3 receptor	Calcitriol	1.40	2001
1LHU	*H. sapiens*	Sex hormone-binding globulin	Estradiol	1.80	2002
1M2Z	*H. sapiens*	Glucocorticoid receptor	Dexamethasone	2.50	2003
1S19	*H. sapiens*	Vitamin D3 receptor	Calcipotriol	2.10	2004
1SQN	*H. sapiens*	Progesterone receptor	Norethisterone	1.45	2004
1SR7	*H. sapiens*	Progesterone receptor	Mometasone furoate	1.46	2004
1UOU	*H. sapiens*	Thymidine phosphorylase	Tipiracil	2.11	2004
1X70	*H. sapiens*	Dipeptidyl peptidase IV	Sitagliptin	2.10	2005
1YI4	*H. sapiens*	PIM-1	Adenosine	2.40	2005
2A15	*M. tuberculosis*	RV0760	Nicotinamide	1.68	2005
2AA6	*H. sapiens*	Mineralocorticoid receptor	Progesterone	1.95	2005
2AM9	*H. sapiens*	Androgen receptor	Testosterone	1.64	2006
2E5D	*H. sapiens*	NAMPT	Nicotinamide	2.00	2007
2F9W	*P. aeruginosa*	Pantothenate Kinase	Pantothenic acid	1.90	2006
2GQG	*H. sapiens*	ABL1	Dasatinib	2.40	2006
2HYY	*H. sapiens*	ABL1	Imatinib	2.40	2007
2P16	*H. sapiens*	Coagulation factor Xa	Apixaban	2.30	2007
2QK8	*B. anthracis*	Dihydrofolate reductase	Methotrexate	2.40	2007
2RIN	*E. coli*	ABC transporter	Acetylcholine	1.80	2008
2W26	*H. sapiens*	Coagulation factor Xa	Rivaroxaban	2.08	2008
2W9H	*S. aureus*	Dihydrofolate reductase	Trimethoprim	1.48	2009
2WGJ	*H. sapiens*	c-Met	Crizotinib	2.00	2009
2XN3	*H. sapiens*	Thyroxine-binding globulin	Mefenamic acid	2.09	2011
2XRH	*H. pylori*	HP0721	Nicotinic acid	1.50	2011
2Y7J	*H. sapiens*	Phosphorylase kinase, gamma 2	Sunitinib	2.50	2011
3APV	*H. sapiens*	Alpha-1-acid glycoprotein 2	Amitriptyline	2.15	2011
3APX	*H. sapiens*	Alpha-1-acid glycoprotein 2	Chlorpromazine	2.20	2011
3AZZ	*T. maritima*	Laminarinase	Gluconolactone	1.81	2011
3B7E	*Influenza A*	Neuraminidase	Zanamivir	1.45	2008
3C7Q	*H. sapiens*	VEGFR2	Nintedanib	2.10	2008
3CSJ	*H. sapiens*	Glutathione S-transferase	Chlorambucil	1.90	2008

3D90	*H. sapiens*	Progesterone receptor	Levonorgestrel	2.26	2009
3EW2	*R. etli*	Rhizavidin	Biotin	2.30	2008
3EYG	*H. sapiens*	JAK1	Tofacitinib	1.90	2009
3F8F	*L. lactis*	LmrR	Daunomycin	2.20	2008
3FL9	*B. anthracis*	Dihydrofolate reductase	Trimethoprim	2.40	2009
3FUP	*H. sapiens*	JAK2	Tofacitinib	2.40	2009
3FUU	*T. thermophilus*	Dimethyladenosine transferase	Adenosine	1.53	2009
3G0B	*H. sapiens*	Dipeptidyl peptidase IV	Alogliptin	2.25	2010
3G0E	*H. sapiens*	KIT	Sunitinib	1.60	2009
3GN8	*H. sapiens*	AncGR 2	Dexamethasone	2.50	2009
3HM1	*H. sapiens*	Estrogen receptor alpha	Estrone	2.33	2010
3I45	*R. rubrum*	Tat pathway signal protein	Nicotinic acid	1.36	2009
3L4W	*H. sapiens*	Maltase-glucoamylase	Miglitol	2.00	2010
3LXK	*H. sapiens*	JAK3	Tofacitinib	2.00	2010
3LXN	*H. sapiens*	TYK2	Tofacitinib	2.50	2010
3MYU	*M. genitalium*	MG289	Thiamin	1.95	2010
3OLL	*H. sapiens*	Estrogen receptor beta	Estradiol	1.50	2010
3QPS	*C. jejuni*	CmeR	Cholic acid	2.35	2011
3QT0	*H. sapiens*	PPAR gamma	Mifepristone	2.50	2012
3RY2	*S. avidinii*	Streptavidin	Biotin	0.95	2011
3SG8	*E. casseliflavus*	APH(2'') IVa	Tobramycin	1.80	2011
3SG9	*E. casseliflavus*	APH(2'') IVa	Kanamycin A	2.15	2011
3SZJ	*S. denitrificans*	Shwanavidin	Biotin	1.45	2012
3TEG	*H. sapiens*	Phenylalanyl-tRNA synthetase	Levodopa	2.20	2011
3TI1	*H. sapiens*	CDK2	Sunitinib	1.99	2012
3U5J	*H. sapiens*	BRD4	Alprazolam	1.60	2011
3U5K	*H. sapiens*	BRD4	Midazolam	1.80	2011
3UE4	*H. sapiens*	ABL1	Bosutinib	2.42	2012
3VHU	*H. sapiens*	Mineralocorticoid receptor	Spironolactone	2.11	2011
3VRI	*H. sapiens*	HLA-A	Abacavir	1.60	2012
3VW1	*S. enterica*	RamR	Gentian violet	2.21	2013
3WAR	*H. sapiens*	CK2 alpha	Nicotinic acid	1.04	2013
4ASD	*H. sapiens*	VEGFR2	Sorafenib	2.03	2012
4BB2	*H. sapiens*	Corticosteroid-binding globulin	Progesterone	2.48	2012
4BBO	*B. japonicum*	Bradavidin	Biotin	1.60	2013
4DT8	*E. casseliflavus*	APH(2'') IVa	Adenosine	2.15	2012
4DVE	*L. lactis*	ECF-type ABC transporter	Biotin	2.09	2012
4E2J	*H. sapiens*	AncGR 2	Mometasone furoate	2.50	2012
4EY6	*H. sapiens*	Acetylcholinesterase	(-)-Galanthamine	2.40	2012
4G1Q	*HIV-1*	Reverse transcriptase	Rilpivirine	1.51	2013
4GCP	*E. coli*	OmpF porin	Ampicillin	1.98	2012
4H2F	*H. sapiens*	Ecto-5'-nucleotidase	Adenosine	1.85	2012
4KS8	*H. sapiens*	PAK6	Sunitinib	1.95	2013
4LZR	*H. sapiens*	BRD4	Colchicine	1.85	2014
4MKC	*H. sapiens*	Anaplastic lymphoma kinase	Ceritinib	2.01	2014
4NMY	*C. difficile*	ABC transporter	Thiamin	1.90	2013
4O0S	*H. sapiens*	Aurora A	Adenosine	2.50	2014
4OAR	*H. sapiens*	Progesterone receptor	Ulipristal acetate	2.41	2014
4P6W	*H. sapiens*	Glucocorticoid receptor	Mometasone furoate	1.95	2014
4P6X	*H. sapiens*	Glucocorticoid receptor	Cortisol	2.50	2014
4QE6	*H. sapiens*	FXR	Chenodeoxycholic acid	1.65	2015
4QMN	*H. sapiens*	MST3	Bosutinib	2.09	2015
4QMS	*H. sapiens*	MST3	Dasatinib	1.88	2015
4QMZ	*H. sapiens*	MST3	Sunitinib	1.88	2015
4QRC	*H. sapiens*	FGFR4	Ponatinib	1.90	2014
4R38	*E. litoralis*	LOV protein	Riboflavin	1.60	2014
4RP9	*E. coli*	UlaA	Ascorbic acid	1.65	2015

4RYA	*A. vitis*	ABC transporter	D-Mannitol	1.50	2014
4S0V	*H. sapiens*	OX2 orexin receptor	Suvorexant	2.50	2015
4TVJ	*H. sapiens*	PARP2	Olaparib	2.10	2015
4U0I	*H. sapiens*	KIT	Ponatinib	2.00	2014
4U95	*E. coli*	AcrB	Minocycline	2.00	2014
4UDA	*H. sapiens*	Mineralocorticoid receptor	Dexamethasone	2.03	2015
4ZN7	*H. sapiens*	Estrogen receptor alpha	Diethylstilbestrol	1.93	2016
4ZOW	*E. coli*	MdfA	Chloramphenicol	2.45	2015
5EDL	*B. subtilis*	ECF transporter	Thiamine	1.95	2016
5G48	*H. pylori*	DNA polymerase III beta	Thiamin	2.28	2017
5I9X	*H. sapiens*	Ephrin A2	Diflunisal	1.43	2016
5KVT	*H. sapiens*	TrkA receptor	Entrectinib	2.45	2017
5NOW	*H. sapiens*	Cyclophilin A	Amifampridine	1.48	2017
5P9I	*H. sapiens*	BTK	Bosutinib	1.11	2017
5TE0	*H. sapiens*	AAK1	Ibrutinib	1.90	2016
5UFS	*H. sapiens*	AncGR 2	Nintedanib	2.12	2017
6VN8	*H. sapiens*	JAK2	Baricitinib	1.90	2021

Table S2. Numerical data showing the TPR values obtained at different distance thresholds, when considering all water molecules. These data complement Fig. 2.

Distance / Å	TPR
0.1	0.026
0.2	0.137
0.3	0.252
0.4	0.371
0.5	0.473
0.6	0.550
0.7	0.624
0.8	0.683
0.9	0.722
1.0	0.751
1.1	0.781
1.2	0.815
1.3	0.835
1.4	0.860
1.5	0.876
1.6	0.899
1.7	0.920
1.8	0.942
1.9	0.964
2.0	0.986

Table S3. Numerical data showing the TPR values obtained at different distance thresholds, when also filtering the crystallographic water sites by EDIA score. These data complement Fig. 3a.

Distance / Å	TPR				
	**EDIA ≥ 0.2**	**EDIA ≥ 0.4**	**EDIA ≥ 0.6**	**EDIA ≥ 0.8**	**EDIA ≥ 1.0**
0.1	0.026	0.027	0.026	0.033	0.038
0.2	0.137	0.141	0.150	0.199	0.269
0.3	0.252	0.260	0.261	0.347	0.474
0.4	0.372	0.381	0.390	0.484	0.641
0.5	0.474	0.481	0.498	0.568	0.731
0.6	0.552	0.558	0.577	0.650	0.833
0.7	0.626	0.633	0.656	0.721	0.872
0.8	0.685	0.693	0.722	0.768	0.885
0.9	0.724	0.732	0.757	0.801	0.897
1.0	0.753	0.762	0.785	0.817	0.923
1.1	0.782	0.792	0.815	0.844	0.923
1.2	0.816	0.823	0.845	0.869	0.949
1.3	0.836	0.843	0.864	0.885	0.949
1.4	0.861	0.866	0.881	0.888	0.949
1.5	0.877	0.880	0.894	0.899	0.949
1.6	0.900	0.902	0.911	0.918	0.949
1.7	0.921	0.921	0.929	0.932	0.949
1.8	0.943	0.944	0.949	0.954	0.974
1.9	0.965	0.967	0.967	0.973	0.987
2.0	0.988	0.988	0.990	0.992	1.000

Table S4. Numerical data showing the TPR values obtained at different distance thresholds, when also filtering the crystallographic water sites by the number of non-water hydrogen bonds. These data complement Fig. 3b.

Distance / Å	TPR			
	≥1 H-bonds	≥2 H-bonds	≥3 H-bonds	4 H-bonds
0.1	0.031	0.053	0.083	0.132
0.2	0.150	0.237	0.331	0.447
0.3	0.278	0.430	0.537	0.737
0.4	0.406	0.567	0.669	0.816
0.5	0.519	0.648	0.760	0.842
0.6	0.594	0.701	0.793	0.868
0.7	0.667	0.754	0.810	0.868
0.8	0.720	0.779	0.826	0.868
0.9	0.761	0.810	0.868	0.921
1.0	0.790	0.847	0.901	0.947
1.1	0.816	0.860	0.917	0.947
1.2	0.846	0.872	0.934	0.947
1.3	0.864	0.888	0.934	0.947
1.4	0.887	0.903	0.950	0.974
1.5	0.900	0.916	0.950	0.974
1.6	0.919	0.931	0.967	0.974
1.7	0.937	0.944	0.967	0.974
1.8	0.956	0.953	0.975	0.974
1.9	0.964	0.963	0.975	0.974
2.0	0.984	0.978	0.992	0.974

Table S5. Numerical data showing the PPV values obtained at different distance thresholds, when also filtering the GCMC water sites by cluster occupancy. Also included are the corresponding data when all GCMC waters are considered. These data complement Fig. 4a.

Distance / Å	PPV				
	All	Occ. ≥ 20 %	Occ. ≥ 40 %	Occ. ≥ 60 %	Occ. ≥ 80 %
0.1	0.003	0.003	0.005	0.007	0.011
0.2	0.015	0.017	0.025	0.037	0.056
0.3	0.027	0.032	0.046	0.068	0.101
0.4	0.040	0.048	0.068	0.099	0.143
0.5	0.050	0.061	0.087	0.126	0.175
0.6	0.059	0.071	0.100	0.146	0.201
0.7	0.067	0.080	0.113	0.164	0.225
0.8	0.073	0.088	0.124	0.180	0.244
0.9	0.077	0.092	0.131	0.187	0.252
1.0	0.080	0.096	0.136	0.194	0.261
1.1	0.083	0.100	0.141	0.200	0.266
1.2	0.087	0.104	0.146	0.206	0.272
1.3	0.089	0.107	0.150	0.211	0.278
1.4	0.092	0.110	0.154	0.216	0.284
1.5	0.093	0.112	0.157	0.220	0.289
1.6	0.096	0.115	0.161	0.226	0.294
1.7	0.098	0.117	0.165	0.230	0.300
1.8	0.101	0.120	0.169	0.236	0.308
1.9	0.103	0.123	0.173	0.241	0.313
2.0	0.105	0.126	0.176	0.246	0.319

Table S6. Numerical data showing the PPV values obtained at different distance thresholds, when also filtering the GCMC water sites by cluster occupancy. These data complement Fig. 4b.

Distance / Å	PPV			
	≥1 H-bonds	≥2 H-bonds	≥3 H-bonds	4 H-bonds
0.1	0.007	0.018	0.038	0.068
0.2	0.035	0.078	0.141	0.23
0.3	0.063	0.141	0.241	0.378
0.4	0.091	0.185	0.293	0.432
0.5	0.116	0.215	0.338	0.473
0.6	0.133	0.233	0.352	0.514
0.7	0.15	0.255	0.362	0.514
0.8	0.162	0.268	0.379	0.514
0.9	0.17	0.279	0.397	0.527
1.0	0.177	0.291	0.414	0.527
1.1	0.182	0.299	0.417	0.527
1.2	0.188	0.306	0.428	0.527
1.3	0.192	0.312	0.428	0.527
1.4	0.197	0.317	0.434	0.541
1.5	0.2	0.323	0.441	0.541
1.6	0.206	0.331	0.445	0.541
1.7	0.209	0.334	0.445	0.541
1.8	0.213	0.339	0.448	0.541
1.9	0.216	0.343	0.448	0.541
2.0	0.22	0.351	0.448	0.541

Supplementary Figures

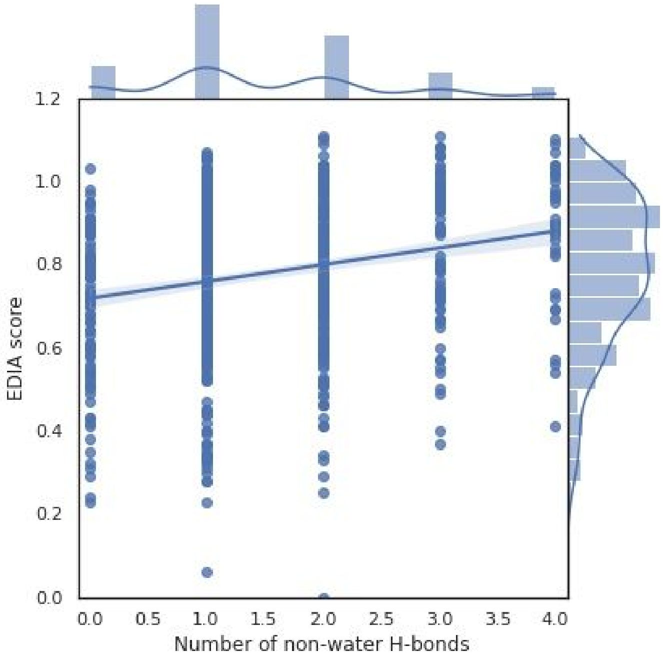

Figure S1. Plot of the number of protein/ligand hydrogen bonds made, against the EDIA score of all 723 crystallographic water sites present in the GCMC boxes of the structures in this work. Also included is a linear regression plot of this data. These data have an R^2 value of 0.05.

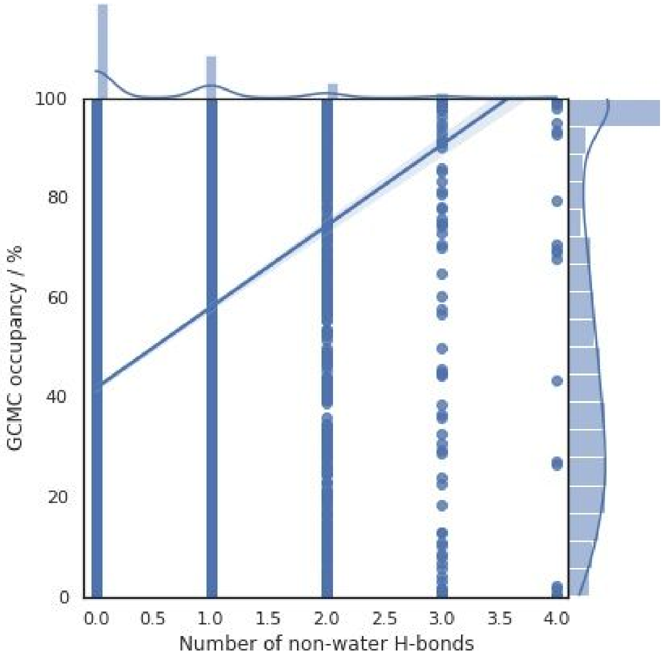

Figure S2. Plot of the number of protein/ligand hydrogen bonds made, against the EDIA score of all 6776 water sites obtained from clustering the GCMC simulation data. Also included is a linear regression plot of this data. These data have an R^2 value of 0.21.
